# Evaluation of Water Intake in Spanish Adolescent Soccer Players during a Competition

**DOI:** 10.2478/hukin-2022-0051

**Published:** 2022-09-08

**Authors:** María del Mar Fernández-Álvarez, Judit Cachero-Rodríguez, Claudia Leirós-Díaz, Sergio Carrasco-Santos, Rubén Martín-Payo

**Affiliations:** 1 Facultad de Medicina y Ciencias de la Salud, Universidad de Oviedo, Oviedo, Asturias, Spain; 2 Equipo de investigación Precam, Instituto de Investigación Sanitaria del Principado de Asturias, Oviedo Spain; 3 Hospital San Agustín, Avilés, Asturias, Spain

**Keywords:** soccer, adolescent, hydration status, water intake

## Abstract

An optimal state of hydration is essential to maintaining health. The objective of this cross-sectional study was to evaluate the water intake of adolescents aged 12 to 16 years and their hydration level during an official soccer match. Three hundred and six players participated in the study (N = 306). Their water intake was recorded and the level of hydration was evaluated using the density of urine as an indicator. Weight measurements were made before and after the match. Water intake control, urine collection and analysis, and the recording of minutes played were carried out after the match. The average weight loss was 746.2 g (SD: 474.07; p < 0.001), with 36.5% with less than 1% loss and 23.3% with more than 2% loss. The mean volume of water ingested was 229.35 ml (SD: 211.11) and a significant correlation was observed between minutes of activity (ρ-value = 0.206; p < 0.001), environmental humidity (ρ-value = - 0.281; p < 0.001), and temperature (ρ-value = 0.200; p < 0.001). The sweat rate was 0.69 l/h (SD: 0.56) and it was significantly associated with playing time (ρ-value = -0.276; p < 0.001). The mean urine density was 1.019 (SD: 0.007), with 64.9% of youth athletes showing dehydration (≥ 1.020). An association was observed between dehydration and activity time (U- value = 4.124; p < 0.001). Approximately 10% of the participants stated that they had not drunk any water during the match. In conclusion, it is necessary to establish individual hydration guidelines based on personal, environmental and activity-related factors, as well as establish a minimum volume of fluids to consume.

## Introduction

Soccer is one of the most practised sports in the world (Arnaoutis et al., 2015). Soccer is characterised by repeated episodes of high-intensity, short-duration runs, alternating with occasional longer-duration runs in a context of high endurance and development of technical and tactical skills (Laitano et al., 2014), causing a high demand for water, e.g. as a result of perspiration caused by a rise in body temperature (Arnaoutis et al., 2015; Da Silva et al., 2012).

Water accounts for 50-70% of body weight, is essential for survival, and plays a fundamental role in different processes, such as cell metabolism, maintenance of electrolyte balance, muscle contraction, and cardiovascular transport of oxygen and nutrients (Sawka et al., 2015). Hence, an optimal hydration status is essential to maintaining health (Armsntrong et al., 2013; Zhang et al., 2017) and developing adequate physical (Kiitam et al., 2018; Michels et al., 2017) and cognitive performance in both adults and children (Kavouras et al., 2016; Sekiguchi et al., 2019; Zhang et al., 2017).

While there is no universal technique for determining hydration status, several methods are recommended in the literature (Armstrong et al., 2016). Urinary biomarkers may determine whether an individual is euhydrated or dehydrated (Armsntrong et al., 2013; Zhang et al., 2017). These biomarkers, in isolation, have considerable limitations (Armstrong et al., 2013). However, when used together in the same context, they can provide very valuable information (American College of Sports Medicine et al., 2007). Urine specific gravity and osmolarity are quantifiable, while colour and volume (Opliger and Bartok, 2002) offer more subjective information (American College or Sports Medicine et al., 2007). Another method is to measure body weight. This method is simple, non-invasive, and is valid for estimating hydration changes in team sports by calculating the difference in pre- and post-activity weight (American College of Sports Medicine et al., 2007; García-Jiménez et al., 2015). Owen et al. (2013) and Sawka et al. (2015) reported dehydration to be a loss of fluid of more than 2% of body weight. A water deficit situation negatively affects aerobic performance, cognitive performance, strength/power development, and technical qualities in soccer (American College of Sports Medicine et al., 2007; Castro-Sepúlveda et al., 2016; García-Jiménez et al., 2015; Sekiguchi et al., 2019). In addition, dehydration increases both the physiological effort and the perception of effort put into performing the same exercise (Arnaoutis et al., 2013).

Analysing the extent to which an individual may be at risk of dehydration and putting in place strategies to prevent it in any age range is, therefore, critical to avoiding the risks associated with dehydration (Marcos et al., 2014). This task is compounded by the large number of factors to consider, such as environmental conditions (Arnaoutis et al., 2013; Hernández-Camacho and Moya-Amaya, 2016; Laitano et al., 2014; Laksmi et al., 2018), clothing, as well as duration and intensity of exercise (Armstrong et al., 2013; Da Silva et al., 2012; Laksmi et al., 2018).

Although the benefits of water and its necessity in humans are well documented, the amount of liquid needed to obtain an appropriate level of hydration seems to be controversial (Eith et al., 2020; Kavouras et al., 2017; Marcos et al., 2014). In this sense, other authors indicate that the amount of fluids and the fluid replacement rate depend on the individual sweat rate, duration of exercise, and opportunities to drink, with sweating being the main route of water loss during exercise (American College of Sports Medicine et al., 2007). Dehydration occurs when fluid loss due to sweating is greater than fluid intake, and is not uncommon when athletes do not drink enough fluids (García-Jiménez et al., 2015; Laitano et al., 2014).

Despite the existence of water consumption guidelines that offer advice for each age group, it is documented that a high percentage of children do not comply with these recommendations (Bougatsas et al., 2018). Different studies have recorded fluid consumption specifically in soccer players. For instance, in the study by Laitano et al. (2014) the liquids consumed by the players were only sufficient to replace approximately 50% of the lost fluids.

Given the benefits that adequate hydration has demonstrated, both in terms of sports performance and cognitive performance in children (Morin et al., 2018), assessment is of paramount importance in determining their needs (Laitano et al., 2014). Existing research has largely focused on measuring data from adult athletes on a single day, with little research including the measurement of data on fluid balance, sweat loss, and hydration status in young soccer players. It is therefore important to investigate the association of these data with fluid intake (Phillips et al., 2014).

In light of the scarce evidence available in this regard, the present study was designed for an adolescent population playing regularly but not professionally soccer in federated teams, with the main objective of assessing fluid intake during the course of an official match and, secondarily, assessing their hydration status.

## Methods

### Participants

This cross-sectional study involved soccer players of both sexes, aged between 12 and 16, who were members of a Spanish soccer federation. First, 363 participants from six soccer teams were selected in September 2018. The legal guardians of participants gave their legal consent in writing. The exclusion criteria were the following: (1) refusal from the minor to have data collected from them; (2) absence or non-participation in the match on the day of the measurement; (3) refusal from the legal representative of the minor to allow them to participate in the study.

Of the 363 players selected, 55 were excluded because they did not play on the day of the evaluations, and further two players were excluded because they refused to have their urine collected on the day of the match. As a result, 306 adolescents (mean age = 13.22; SD: 1.08) agreed to participate in the study, with 91.8% being male (*n* = 281) and 8.2% female (*n* = 25). The mean environmental temperature and relative humidity during data collection, measured with OREGON SCIENTIFIC BAR-206, were 11°C (range: 3.8-18.8; SD: 4.76) and 72.9% (range 40-93.5; SD: 14.06), respectively.

The measurements and recording of the different variables were carried out before and after an official soccer match. Players were summoned at the sports facilities 90 minutes before the start of the match. Inside the locker room, the protocol to be carried out was explained to players, i.e., weighing before and after the match, the procedure for collecting a post-match urine sample, and the importance of remembering the amount of water they would ingest from the warm-up to the end of the match. The containers for the collection of urine samples were previously tagged with the alphanumeric code identifying each player and deposited in a box. Players were asked to leave the urine sample in that box after the post-match urination. The environmental temperature and relative humidity at the beginning of the warm-up and at the end of the match were also recorded.

The study was designed in accordance with the Declaration of Helsinki and was approved by the Ethics Committee.

### Measurements

Weight measurements were made before and after the match. Water intake control, urine collection and analysis, and the recording of minutes played were carried out after the match.

In order to record body weight, players, dressed in competition clothing (trousers, shirts, socks, and underwear, exclusively) were placed barefoot on the centre of the weighing scales, distributing their weight between their feet, facing forward, with their arms along the body, and without making any movements. The result which appeared on the screen of the scales was recorded on the corresponding log sheet.

Prior to the post-match body weight collection, players urinated into a sterile container identified by their personal code. Once the urine was placed in the corresponding box, players were weighed following the same pre-match weighing protocol. Once done, players were asked about the amount of liquid ingested from the warm-up to the end of the match. The intake was ad libitum. All players had bottles of water at their disposal to drink whenever they needed to, in accordance with their usual match routine.

### Urinalysis

The sample was analysed using the ONE+STEP DUS10 Health Mate urine test strips. The test strips were inserted completely into the sterile sample collection cups for 2 s and then read after additional 60 s, strictly following the protocol indicated by the instructions. The results were obtained by direct comparison with the colour chart printed on the tube containing the strips. The results were recorded on the corresponding log sheet. As with other authors, < 1.020 g/l was considered to be the accepted cut-off point for euhydration (Phillips et al., 2014).

### Statistical Analysis

A descriptive study of the variables was carried out using the indices typical of descriptive statistics: absolute frequencies, percentages, means, and typical deviations. In addition, a normality analysis of the sample was performed using the Kolmogorov-Smirnov test.

To test the quantitative variables, the Mann-Whitney *U*-test and the Spearman’s rank correlation test were used. *χ*-squared was used for qualitative variables. For all cases, the results were considered to be statistically significant when *p* ≤ 0.05. Data were analysed using SPSS software, version 24.0.

## Results

### Minutes of activity, body weight, weight and sweat lost

The average time of activity was 81.40 minutes (SD: 25.50; 95% CI: 78.44-84.36) similar in both sexes (*p-value* = 0.574). The mean body weight before the match was 51.6 kg (SD: 11.75; 95% CI: 50.28-53.01) and, after the match, 50.9 kg (SD: 11.61; 95% CI: 49.55-52.25), which yielded a mean weight loss of 746.2 g (SD: 474.07; 95% CI: 690.98-801.33; *p* < 0.001). The percentage of weight lost was 1.45 (SD: 0.90; 95% CI: 1.35-1.56), with 36.5% of participants having lost less than 1% of their weight, and 23.3% of participants having lost more than 2% of their weight. No significant differences between sexes were observed for any of these variables.

### Water intake

Overall, the mean volume of water drunk by the players was 229.35 ml (SD: 211.11; 95% CI: 204.87-253.84). Approximately 10% of the participants stated that they had not drunk any water during the match. The volume of water consumed by boys (mean = 226.52; SD: 213.27; 95% CI: 200.77-252.26) was slightly lower than that consumed by girls (mean = 263.64; SD: 183.83; 95% CI: 182.13-345.14). However, this difference was negligible (*p* = 0.167). A significant correlation was observed between minutes of activity (*p value* = 0.206; *p* < 0.001), relative humidity (*p value* = - 0.281; *p* < 0.001), and environmental temperature (*p value* = 0.200; *p* < 0.001).

**Table 1:** Sex differences in pre- and post-match weight and weight lost

	Male	Female	*p*
Mean pre-match weight, kg (SD; 95% CI)	51.79 (11.99;50.33-53.24)	49.97 (8.31; 46.29-53.66)	0.709
Mean post-match weight, kg (SD; 95% CI)	51.02 (11.86; 49.59-52.46)	49.40 (8.28; 45.72-53.07)	0.751
*p*	<0 .001	<0 .001	
Mean weight loss, gr (SD; 95% CI)	760.23 (481.97; 760.23-818.63)	577.27 (329.40; 431.22-723.32)	0.108
% weight lost (SD; 95% CI)	1.48 (0.91; 1.37-1.59)	1.17 (0.66; 0.88-1.47)	0.160
Mean sweat rate, l/h (SD; 95% CI)	0.99 (0.52; 0.92-1.05)	0.84 (0.38; 0.68-1.01)	0.395

**Table 2:** Relationships between urine density (split by hydration status: hydration vs. dehydration) and weight loss (g), water intake (ml), sweat rate (in l/h), and duration of activity (min)

	Hydration	Dehydration	*U-value*	*p*
Mean weight loss; SD	707.53; 488.84	771.10; 473.46	1.162	0.245
(95% CI)	(606.85-808.20)	(700.05-842.15)		
% mean weight loss; SD	1.35; 0.88	1.50; 0.89	1.365	0.172
(95% CI)	(1.17-1.53)	(1.37-1.63)		
Mean water intake; SD	203.58; 213.48	240.72; 204.90	1.905	0.057
(95% CI)	(159.62-247.55)	(209.97-271.47)		
Sweat rate; SD	0.94; 0.75	0.76; 0.51	1.493	0.136
(95% CI)	(0.79-1.10)	(0.69-0.84)		
Mean total duration of activity; SD	70.38; 28.68	86.84; 22.32	4.124	< 0.001
(95% CI)	(64.47-76.28)	(83.49-90.19)		

The sweat rate was 0.69 l/h (SD: 0.56; 95% CI: 0.62-0.75). Again, a significant association was observed between this variable and playing time (*p value* = -0.276; *p* < 0.001). However, no significant association was observed with environmental conditions or personal variables. The percentage of liquid replaced was 44.20% (SD: 96.04; 95% CI: 33.02-55.38).

### Urine density

The mean urine density was 1.019 g/L (SD: 0.007; 95% CI: 1.019-1.020). No significant differences between sexes were observed (*p* = 0.603), nor were they observed on the bases of environmental temperature (*p* = 0.186) or relative humidity (*p* = 0.389). On the basis of urine density, 35.1% of participants had a normal hydration status (≤ 1.019) versus 64.9% who were dehydrated (≥ 1.020). No relationship was observed between the level of hydration and weight or water consumption, but there was a relationship with the duration of the activity.

## Discussion

The results of the present study show that there is a high percentage of adolescents with low water consumption and a high percentage of adolescents experience dehydration during and after soccer practice. A clear relationship was found between environmental factors, temperature and humidity, and the duration of physical activity and water consumption. In addition, it was verified that the variable that best predicted the risk of dehydration was duration of activity.

The undeniable benefits of water, described by some authors as the liquid that contributes to better hydration (Bougatsas et al., 2018), and its popularity among children and adolescents (Iglesia et al., 2016) make it a recommendable liquid to consume during physical activity. The mean consumption of water in the present study, approximately 230 ml/adolescent, was much lower than the consumption described by other authors in previous studies (Hernández-Camacho and Moya Amaya, 2016; Phillips et al., 2014). In addition, it should be noted that approximately 10% of the adolescents said they had not consumed any water during their activity. In the present study, the amount of liquid ingested was not found to influence the adolescent’s post-match hydration levels, an effect previously found and documented by other authors (Hernández-Camacho and Moya-Amaya, 2016). However, that amount should still be considered, because its effects may appear after a long time. In general, there seems to be a trend towards low fluid intake in both adolescents in Spain (Iglesia et al., 2016) and adolescents in other countries (Michels et al., 2017; Suh and Kavouras, 2018), regardless of physical exercise, which is harmful to health. Specifically, in the field of sports and to avoid this, Cleary et al. (2012), Duffield et al. (2012) and Owen et al. (2013) have suggested establishing a pattern of water consumption as an effective measure to prevent the occurrence of dehydration.

Another result showed the relationship between water consumption and weather conditions, as in previous studies (Mohr et al., 2012; Sekiguchi et al., 2019). There is an abundance of literature which documents that during sport, there are direct relationships both between environmental temperature and fluid intake (Mohr et al., 2012), and between temperature and sweat secretion (Sawka et al., 2015). Nonetheless, no relationship was observed between weather conditions and the players’ dehydration, in contrast with other authors who did provide evidence of such a relationship. As a consequence, it is worth taking environmental conditions into account when planning for the euhydration of athletes (Sawka et al., 2015). The absence of association observed in the present study should be regarded with some caution, as the weather conditions were not extreme, which may help explain the results. It is also well known that weather conditions influence the sensation of thirst. As a result, if weather conditions do not create the need to drink, adolescents will not drink. As Arnaoutis et al. (2013) pointed out, drinking solely on the basis of the sensation of thirst poses a risk of dehydration.

Drawing on the literature reviewed (Opliger and Bartok, 2002; Sawka et al., 2015), it was decided to determine the level of hydration by observing the density of urine and the percentage of weight lost from the beginning to the end of the activity. Approximately 65% of youth athltes presented urine density values compatible with dehydration at the end of the activity. Previous studies assessing dehydration in children, in the absence of physical activity, found a clearly lower percentage of children with such urine density values (Eith et al., 2020), which reveals an increased risk of dehydration in children during soccer practice. In this sense, it is also worth noting that the mean weight loss, as a percentage of overall weight, was 1.45% (SD: .90; 95% CI: 1.35-1.56). Furthermore, 63.5% of the participants lost more than 1% of their body weight. None of these variables were influenced by the amount of liquid ingested, a circumstance which was documented in previous studies (Laitano et al., 2014). For instance, similar research carried out in soccer players, in which participants consumed a greater amount of liquid, showed lower (Phillips et al., 2014) or greater (Da Silva et al., 2012) losses. This inconsistency must therefore be attributed to other variables, or at least not exclusively to fluid intake.

This statement is further reinforced by the observed relationship between dehydration and the duration of the activity. The duration of physical activity has been highlighted as the best predictor of dehydration in the population studied with a direct relationship: the greater the duration of activity, the greater the degree of dehydration. García-Jimenez et al. (2015) observed this association. However, as in our case, they pointed to the need to take into account other factors, such as environmental ones. In this sense, several authors have recommended taking into account the characteristics of exercise in order to schedule an adequate degree of fluid intake and thus prevent dehydration (Da Silva et al., 2012; Phillips et al., 2014).

Additionally, the duration of the activity was the variable that was related to the sweat rate. Duffield et al. (2012) indicated that the sweat rate was influenced by the intensity of physical activity. Given that participants in this study carried out, in similar categories, the same activity, it is fairly safe to say that the activity time variable may be assimilated into intensity and, therefore, the results are consonant. It is important to note that, as it was the case for fluid intake, no relationship was observed between the sweat rate and urine density at the end of the match. This has also been previously documented by other authors (Da Silva et al., 2012; García-Jiménez et al., 2015), which does not prevent it from being taken into account as a consumption estimator in the absence of a more effective indicator. Again, as indicated in previous studies, other factors must be considered in each particular context that specifically help explain the sweat rate figures, such as environmental temperature (Laitano et al., 2014; Mohr et al., 2012). On a negative note, another aspect worth highlighting from the results of the present study is the low percentage of liquid replaced, approximately 38%. This is clearly insufficient to meet the needs of players and is lower than the percentages observed by other researchers (García-Jiménez et al., 2015; Phillips et al., 2014).

One possible limitation to take into consideration is that the hydration level had not been measured prior to the start of the match. However, the main objective of the study, which was to assess the players’ fluid intake during physical activity, has indeed been carried out. In addition, the measurement of the hydration level was taken into consideration, bearing in mind that, as several authors point out, it is not exclusively linked to the amount of liquid ingested during physical activity (American College of Sports Medicine et al., 2007; Sawka et al., 2015). Future research should be carried out in regions with extreme climatic conditions, which can contribute to assessing the real needs for fluid intake in populations with similar characteristics.

## Conclusions

It is necessary to establish individual hydration guidelines based on personal, environmental, and activity-related factors. In addition, it is also necessary for those in charge of the minors to encourage the intake of liquids by establishing a minimum volume of fluids to consume, as opposed to ad libitum consumption, and by facilitating access to liquids according to environmental measurements.
